# Catecholaminergic Modulation of Semantic Processing in Sentence Comprehension

**DOI:** 10.1093/cercor/bhaa204

**Published:** 2020-08-08

**Authors:** Yingying Tan, Peter Hagoort

**Affiliations:** 1 Max Planck Institute for Psycholinguistics, Nijmegen 6525 XD, The Netherlands; 2 Donders Institute for Brain, Cognition, and Behaviour, Radboud University, Nijmegen 6500 HB, The Netherlands

**Keywords:** catecholamine, language processing, late positive complex (LPC), methylphenidate, N400

## Abstract

Catecholamine (CA) function has been widely implicated in cognitive functions that are tied to the prefrontal cortex and striatal areas. The present study investigated the effects of methylphenidate, which is a CA agonist, on the electroencephalogram (EEG) response related to semantic processing using a double-blind, placebo-controlled, randomized, crossover, within-subject design. Forty-eight healthy participants read semantically congruent or incongruent sentences after receiving 20-mg methylphenidate or a placebo while their brain activity was monitored with EEG. To probe whether the catecholaminergic modulation is task-dependent, in one condition participants had to focus on comprehending the sentences, while in the other condition, they only had to attend to the font size of the sentence. The results demonstrate that methylphenidate has a task-dependent effect on semantic processing. Compared to placebo, when semantic processing was task-irrelevant, methylphenidate enhanced the detection of semantic incongruence as indexed by a larger N400 amplitude in the incongruent sentences; when semantic processing was task-relevant, methylphenidate induced a larger N400 amplitude in the semantically congruent condition, which was followed by a larger late positive complex effect. These results suggest that CA-related neurotransmitters influence language processing, possibly through the projections between the prefrontal cortex and the striatum, which contain many CA receptors.

## Introduction

Methylphenidate (MPH; Ritalin) is the most commonly prescribed drug for attention deficit hyperactivity disorders (ADHD) because of its efficacy and safety (Goldman et al. 1998; Volkow and Swanson 2003; Linssen et al. 2014). It is an indirect catecholamine (CA) agonist that increases the extracellular levels of dopamine (DA) and noradrenaline (NA) in the brain by blocking their reuptake (Volkow et al. 1998, 2001, 2004; Andrews and Lavin 2006; Clatworthy et al. 2009; Hannestad et al. 2010). The prefrontal cortex (PFC) and striatum contain a large number of CA receptors, and thus, the administration of MPH has been demonstrated to have effects on cognitive functions that are closely tied to the PFC and striatal networks (for reviews, see Leonard et al. 2004; Repantis et al. 2010; Cools and D’Esposito 2011). Evidence from both neuropsychological studies in patients with CA-related dysfunctions (e.g., Parkinson’s disease [PD], ADHD, schizophrenia) and pharmacological studies in the healthy population (e.g., administering CA agonists such as MPH, L-dopa, or amphetamine) have demonstrated that MPH facilitated many cognitive functions, including the speed of processing, attention, response inhibition, working memory (WM), learning, decision making, creativity, and language processing (Mehta et al. 2000; Arnsten and Dudley 2005; Repantis et al. 2010; Tye et al. 2010; Smith and Farah 2011; Tomasi et al. 2011; Agay et al. 2014; Carmack et al. 2014; Moeller et al. 2014; Ter Huurne et al. 2015; Rosenberg et al. 2016; Boot et al. 2017). Previous pharmacological functional magnetic resonance imaging (fMRI) studies have shown that MPH exerts its influence on cognitive functions through modifying brain activities in the PFC and the striatum, while the precise neural site of modulation depends on the nature of the cognitive process (Dodds et al. 2008; Fallon et al. 2017).

However, the exact effect of MPH on cognitive functions remains controversial. A general conclusion drawn from the previous studies is that there is no overall enhancing effect of MPH (see a review from Smith and Farah 2011). A number of studies even observed a detrimental effect of MPH on certain cognitive functions, such as flexible updating (Mehta et al. 2000; Dodds et al. 2008; Fallon et al. 2017) and recognition memory (LeBlanc-Duchin and Taukulis 2007). Reviews on the MPH effects in patients, healthy individuals, and nonhuman animals suggest that the variability in MPH effects may be explained by a number of factors, such as the dosage of MPH (Arnsten and Dudley 2005; Cooper et al. 2005), the nature of the tasks (Elliott et al. 1997; Dodds et al. 2008), and individuals’ baseline levels of performance (Elliott et al. 1997; Mehta et al. 2000; Dodds et al. 2008; Clatworthy et al. 2009; Van der Schaaf et al. 2013). For example, in line with the finding that the relationship between DA level and individual’s performance on cognitive tasks follows an inverted-U-shaped function (Cools and D'Esposito 2011), there were also studies demonstrating that MPH produced an inverted-U-shaped response. Several studies have shown that low or moderate doses of MPH significantly improve cognition, whereas high doses cause perseverative errors (Arnsten and Dudley 2005; Arnsten 2006).

In addition, a number of studies have shown that CA agonists have a task-dependent effect. The CA agonists have been found to enhance rewarding or task-relevant behavior while inhibiting nonrewarding or task-irrelevant behavior (Volkow et al. 2001; Durstewitz and Seamans 2008; Ter Huurne et al. 2015). Such task-dependent effects of CA agonists might be related to the well-studied role of DA neurons in processing rewarding and nonrewarding events in opposite directions. Many studies have shown that DA neurons are excited by task-relevant signals while inhibited by task-irrelevant signals (see Durstewitz and Seamans 2008 for a review). However, it has become increasingly clear that some DA neurons are also excited by intrinsically nonrewarding but salient or even aversive events (Volkow et al. 2001, 2004; Bromberg-Martin et al. 2010; Ter Huurne et al. 2015). Results from animal models have demonstrated that some DA neurons are excited by both rewarding and salient events, whereas they show weaker responses to neutral events. This is crucial for adaptive behavior as it allows neural circuitry to respond to events of high importance (see a review by Bromberg-Martin et al. 2010). In the human studies, Ter Huurne et al. (2015) also found that instead of inhibiting task-irrelevant processing, MPH enhanced the processing of distractors, when the distractor and target were from the same category (e.g., both target and distractor are faces). This actually led to impaired processing of the target. The implication from these studies to our current study is that by administrating MPH, we expected to observe changes in individual’s behavior in processing both rewarding/task-relevant and nonrewarding/task-irrelevant but salient events. It has been reported that the effect of CA agonists depends on individuals’ baseline levels of performance, such as WM capacity. Individuals with poor WM capacity showed greater beneficial effects of CA agonists, while individuals with high WM capacity performed worse after receiving MPH (Kimberg et al. 1997; Mehta et al. 2004; Agay et al. 2014; Rosenberg et al. 2016).

So far, few pharmacological studies have examined the MPH effect on language processing in the healthy population, although previous neuropsychological studies have provided strong evidence that CA plays a crucial role in language processing (Angwin et al. 2006, 2009, 2017; Andreou et al. 2014; McNamara and Durso 2018). Several pharmacological studies using other CA agonists (e.g., L-dopa, pergolide) have demonstrated that CA agonists enhance direct or high-frequency semantic priming (e.g., “bank–money”) while inhibiting indirect (e.g., “summer–snow,” as “summer” primes “winter” then “winter” primes “snow”) or low-frequency semantic priming (e.g., “bank–river,” as “river” is related to the subordinate meaning of “bank”) at the word level (Kischka et al. 1996; Copland et al. 2003, 2009; Angwin et al. 2004). Copland et al. (2009) showed that the CA agonist exerts this frequency-based modulation effect on semantic priming by influencing the activation of PFC, temporal lobe, and anterior cingulate. This finding was consistent with considerable neuroimaging evidence that the cortico-subcortical connectivity between the striatum and the PFC plays a crucial role in language processing (Snijders et al. 2010; Xiang et al. 2010; Hagoort 2013, 2017).

In the present study, we aimed to examine the effect of MPH on semantic processing during sentence comprehension in combination with an electroencephalogram (EEG) recording. We addressed this issue through exploring how MPH modulates the N400 response, which is a well-established event-related potential (ERP) related to semantic processing (Kutas and Hillyard 1980; see Kutas and Federmeier 2011 for a review). N400 is a negative deflection that occurs approximately 250–500 ms after word onset with a centro-posterior scalp distribution and a peak at around 400 ms. A reliable and consistent finding from previous research is that the amplitude of the “N400 response” was proportional to the difficulty of semantic processing, reflecting a continuous pattern of neuronal activity of accessing and integrating the incoming words (Federmeier et al. 2007; Baggio and Hagoort 2011; Brothers et al. 2015). We refer to the amplitude differences of the N400 response between different semantic conditions as the “N400 effect.” In addition to the N400 effect, we were interested in the late positive complex (LPC) (Some researchers suggested that there were differences between LPC and syntactic P600, as the LPC reflects a deeper or extended processing, while P600 was associated with syntactic reanalysis or repair (Kos et al. 2012). In the current project, we used the term of LPC and did not aim to distinguish between LPC and P600, which was not the focus of the current study.), which has been related to more elaborate or deeper processing during language comprehension (Osterhout and Holcomb 1992; Münte et al. 1998; Van Herten et al. 2006; Van de Meerendonk et al. 2010; Stroud and Phillips 2012; Leckey and Federmeier 2020). The LPC is a positive deflection that occurs approximately 500–550 ms after critical word onset and lasts until at least 800 ms, with a broad central–posterior scalp distribution. Previously, a biphasic N400-LPC effect was observed while participants were reading semantically anomalous but grammatically well-formed sentences (Swick et al. 1998; Van Petten and Luka 2006, 2012; Kos et al. 2012). Therefore, the LPC has been related to the possible cost of processing unexpected words. Researchers suggested that LPC reflects a general attentional control or conflict monitoring process (see Stroud and Phillips 2012, for a review). However, it should be noted that the N400 effect was not always followed by a LPC effect (Osterhout and Mobley 1995; Van den Brink et al. 2006; Van de Meerendonk et al. 2010; Kos et al. 2012).

## The Present Study

The central question of this study is whether and if so, to what extent, CA has an influence on language processing. The current study used MPH as a proxy of the CA influence and examined the modulation effects of MPH on ERPs associated with semantic processing, in a double-blind, placebo-controlled, randomized, crossover, within-subject design. First, we predicted to replicate the N400 effect elicited by the semantically incongruent versus congruent sentences regardless of task requirements or drug administration. Second, we expected to observe a neuropharmacological effect of MPH on language processing. Moreover, we hypothesized that such effect might be task-dependent. That is, MPH will enhance semantic processing when it is task-relevant, as indexed by a larger N400 effect. There might also be a larger LPC effect after receiving MPH as participants may exert more attentional control on semantic processing. Overall, we suggested that CAergic system could up- and downregulate language processing, through modifying the saliency of language processing through the striatum to the prefrontal cortex projections. In addition, we conducted two exploratory analyses: first, we examined whether the MPH effects were mediated by individuals’ baseline levels of performance through relating the size of the MPH effects to individuals’ working memory capacity, language proficiency, and general processing speed. Second, based on visual inspection of our EEG data, for exploratory purposes, we conducted a post hoc analysis on the MPH effect in the pre-N400 time window to see if there were any early influences of the CAergic system on language processing.

### Materials and Methods

#### Participants

Forty-eight healthy native Dutch speaker (20 male; age range, 19–30 years; mean age, 22.1 years, SD = 2.4) participated in this experiment. All participants were right-handed and had normal hearing, motor control, and normal or corrected-to-be normal vision. None of the participants had a major history of neurological or psychiatric disorder. All participants gave a written consent form before entering the experiment. They were carefully screened by a responsible physician from Radboud University Medical Center before starting the experiment. Participants were required to abstain from alcohol and smoking for 24 h and from psychotropic medication or recreational drugs for 72 h prior to testing. A light breakfast without caffeinated drink was allowed, and light snacks were provided during the testing. Participants were compensated with 100 Euro. Six participants were excluded from the statistical analysis due to excessive artifacts in the EEG signals. Therefore, 42 participants were included in the data analysis (18 male; age range, 19–28 years; mean age, 23.3 years, SD = 2.2). This study was conducted according to the Good Clinical Practice guidelines and was approved by the local ethics committee of the Netherlands (NL51075.091.14).

### Pharmacological Design

This study used a double-blind, placebo-controlled, randomized, crossover, within-subject design. Each participant was tested in two sessions with at least 1 week apart (mean = 8.8 day, SD = 3.3) to insure drug washout. Half of the participants received an oral capsule of immediate-release 20-mg MPH in session 1 and an identically overcoated placebo in session 2, while the other half took the capsules in reversed order. The recommended dosage for ADHD treatment is 20 mg regardless of body weight (Sunohara et al. 1999). We opted for this dose to minimize potential risks. The dose of 20-mg MPH has been shown to be sufficient to affect cognitive functions such as cognitive control and reversal learning (Clatworthy et al. 2009; Repantis et al. 2010; Van der Schaaf et al. 2013) and was even found to show a comparable effect as a higher dose of 40-mg MPH on response inhibition (Linssen et al. 2012). In each session, the testing was conducted in the following order: 1) before capsule administration, participants were tested on a set of baseline measures for their cognitive abilities (about 40 min; see Baseline Levels of Performance, Physical Symptoms, and Subjective Mood section for details); 2) the capsule administration was followed by an approximately 60-min waiting period for MPH to reach its maximal plasma level; 3) about 60 min after capsule administration, participants were tested on three short cognitive ability tests (see Baseline Levels of Performance, Physical Symptoms, and Subjective Mood section for details), and their spontaneous eye blink rate (SEBR) was calculated from a 5-min recording. Participants were instructed to look at a fixation cross presented at the center of the screen and blink naturally, while their vertical EOG was recorded from the electrodes FP1/2 mounted on an EEG cap and an additional electrode placed below the left eye. Some previous neuropsychological studies have demonstrated that SEBR is a good clinical marker indexing DA receptor availability (Taylor et al. 1999; Groman et al. 2014), and thus, it could serve as an indirect measurement for individual DA levels; 4) the sentence comprehension task was carried out about 90 min after capsule administration. Dose selection and timing of testing were based on previous neuropharmacological data (Volkow et al. 1998; Clatworthy et al. 2009; Van der Schaaf et al. 2013; Ter Huurne et al. 2015). In addition, participants’ blood pressure and heart rate were monitored during the experiment.

### Sentence Comprehension Experiment

Participants were instructed to read sentences silently on the screen and then answer a question following each sentence. The stimuli consisted of 360 Dutch sentence pairs, which were selected and modified from a set of stimuli that has been used in previous studies and were known to reliably elicit a N400 effect (Van Den Brink et al. 2001; Hagoort 2003; Hagoort et al. 2004; Kos et al. 2012). In each sentence pair, the two sentences were matched on all the words except the adjective–noun phrase, resulting in a semantically congruent and an incongruent condition, e.g., “De slimme/^*^zoute studenten geven een lezing op het congress” (English translation: “The smart/^*^salty students gave a lecture at the congress”). The average length of the experimental sentences was 10.2 words (SD = 2.5), and the position of the critical noun was never in the sentence final position. To avoid participants developing a predictive processing strategy, the position of the critical noun varied (mean = 6.4, SD = 3.1, range = 3rd to 14th). The critical nouns were matched for the number of syllable and word frequency as computed in the SUBTLEXus database (Brysbaert and New 2009) across the congruent and incongruent conditions. To avoid the repetition of the sentence content within a participant, the entire stimulus set of 720 sentences was randomly assigned into two different lists with the two variants from each pair distributed over the two lists.

During the experiment, the sentences were presented using a rapid visual serial presentation. Each sentence started with a fixation cross appearing in the center of the screen for 500 ms, and each word was presented for 300 ms followed by a blank screen for 300 ms. The final word of each sentence was presented with a period. Then a question was presented after the final word. Two task requirements were added since it is known that these can affect the processing salience of the language input (Chwilla et al. 1995). These task requirements led to two types of questions to compare MPH effects on purposeful semantic processing and involuntary semantic processing: 1) in the “Semantic” task, participants were instructed to read the sentence carefully for comprehension. After each sentence, a comprehension question was presented in the center of the screen asking “Vond je deze zin plausibel?” (English translation: Did you find this sentence plausible?) Participants were asked to answer this question by pressing the left or the right button on a button box. “Yes” or “No” buttons were counterbalanced across participants as for half of the participants, the left button was associated with a Yes answer, and the right button was associated with a No answer, while for the other half, the association was reversed; 2) in the “Font-size” task, participants were told that they needed to compare the font size of the sentential words with a following probe word, which was presented after the sentence’s final word and was semantically unrelated to the sentence context. In half of the trials, the probe word was presented in the same font size as the sentence words in a white lowercase Arial 18-point font size. In the other half of the trials, half of the probe words were presented in a larger font size (mean = 21.1 points, SD = 0.8 points), while the other half were presented in a smaller font size (mean = 16.0 points, SD = 0.8 points). These font sizes were selected to match the task difficulty between the Semantic and the Font-size tasks as much as possible. A separate group of native Dutch speakers (*N* = 16), who did not participants in later EEG experiment, were recruited for a behavioral test with the exactly same sentence materials. The Semantic and the Font-size tasks were matched on task difficulty in this pilot study (*d*-prime (*d′*): *t*(15) = 0.61, *P* = 0.55) (After testing several participants on the Font-size judgment task only, we adjusted the font-size selection and tested a separate group of 16 native Dutch speakers on the experimental materials in a self-paced reading experiment. The Semantic and Font-size tasks were matched on difficulty (*d′*: *t*(15) = 0.61, *P* = 0.55). However, participants responded faster to the semantic judgment questions than the font-size judgment questions (739 vs. 932 ms), *F*(1, 15) = 13.82, MSE = 0.27, *P* = 0.002. This RT difference could be explained by the fact that semantic violations could be detected during online reading and participants were ready for the button press even before finishing reading the sentence, while they have to wait for and process the probe word which was presented after the sentence final words in the Font-size judgment task. This difference was expected in our current design but would have little influence on the interpretation of the main online EEG results.). During the experiment, the order of the tasks was kept consistent between the two sessions for each participant and counterbalanced across participants. To prevent item-specific effects, sentences in the Semantic and the Font-size tasks were counterbalanced as each sentence appeared in different condition across the two lists. Twelve practice trials were presented before each session to get participants familiar with the test. The experiment lasted about 30 min.

### Baseline Levels of Performance, Physical Symptoms, and Subjective Mood

Participants’ baseline levels of performance was tested on a set of widely used individual difference measures before capsule administration, including two WM tasks (i.e., automatic reading span and operation span) (Daneman and Carpenter 1980; Unsworth et al. 2005), a language proficiency task (Neger et al. 2015), a perceptual speed task (i.e., box completion) (Salthouse 1993), and an ADHD DSM-IV rating scale (DuPaul et al. 1998; Kooij et al. 2005). In addition, to control for unspecific MPH effects on arousal and attention, several cognitive tasks were included: 1) three short individual differences tests, including a visual attention test (i.e., number cancellation) (Moran and Mefferd Jr 1959) and two short versions of working memory tasks (i.e., operation span and symmetry span, each lasted about 7 min) (Foster et al. 2015), and 2) a visual analogue scale (VAS) (Bond and Lader 1974) assessing participants’ subjective mood, including 16 questions (e.g., alert–drowsy, muzzy–clear-headed). Their physical symptoms were examined via 10 questions about physical complaints (e.g., headache, dry mouth). In both tasks, participants were instructed to move the mouse on the screen on a continuous scale between opposite ends of each dimension to indicate their answer. The VAS and the physical symptom questionnaires were conducted at two time points, 1) approximately 1 h before capsule administration and 2) approximately 1 h after capsule administration; 3) participants’ cardiovascular parameters (i.e., blood pressure and heart rate) were monitored four times during the experiment. The average of the first two measurements (~1 h before and immediate before capsule administration) and the last two measurements (~1 and 3 h after capsule administration) were calculated for examining the cardiovascular effect of MPH.

### Electroencephalogram (EEG) Data Acquisition and Preprocessing

The EEG was recorded from 26 Ag/AgCl electrodes mounted in a customized cap (actiCAP) according to the international 10–20 system. Bipolar horizontal EOG was recorded from two additional electrodes placed at the outer left and right canthi. Vertical EOG was recorded from FP1/2 on the cap and an additional electrode placed below the left eye. The ground electrode was placed on the forehead. In addition, two electrodes were placed on the left and the right mastoids. During the recording, all electrodes were referenced to the left mastoid, and their impedances were kept below 15 kΩ. The EEG and EOG signals were amplified through a BrainVision DC amplifier with a 200-Hz low-pass filter and digitized online with a 500-Hz sampling frequency.

The EEG data was processed using the open-source EEGLAB (Delorme and Makeig 2004) and FieldTrip toolbox (Oostenveld et al. 2011). A bandpass filter of 0.1–30 Hz was applied, and the data were re-referenced offline to the average of the left and right mastoids. The eye movements were corrected by independent component analysis (ICA) (Jung et al. 2000). Then the continuous data were segmented into epochs of −150 to 1200 ms time-locked to the onset of the critical noun. An averaged prestimulus baseline of 150 ms was used. Only trials with a correct response were included. Trials contaminated by artifacts, such as excessive muscle activity, eye movements that were not corrected after applying ICA, were removed following standard procedures (Lopez-Calderon and Luck 2014). Any trial with a mean voltage exceeding ±100 μV or a peak-to-peak amplitude exceeding 100 μV was rejected. This resulted in the exclusion of approximately 14% of the raw data.

### Analysis

#### Behavioral Data

To measure the influence of MPH on participants’ sensitivity to semantic and perceptual incongruence, *d′* was calculated based on accuracy to the comprehension questions and analyzed by repeated measures ANOVAs with the factors of MPH (MPH vs. placebo) and Task (Semantic vs. Font-size task) as within-subject variables and Order (the order of drug administration: MPH-placebo vs. placebo-MPH) as a between-subject variable. Among the 42 participants, 22 of them took MPH in session 1 and placebo in session 2 (Order 1), while the other 20 took the reversed order (Order 2). The Order factor was included to examine the potential interaction of practice-induced differences between sessions and MPH effect in a within-subject design, because previous studies have shown that repeated practicing or familiarization improves individuals’ language comprehension performance (Herman 1985; Rugg 1985; Snijders et al. 2007). Then the reaction time (RT) data was analyzed by 2(MPH) × 2(Task) × 2(Semantic congruence) × 2(Order) repeated measures ANOVAs.

#### Event-Related Potentials

Averaged ERPs on the critical word (i.e., the noun) were computed for each condition and each subject separately. Based on the prior knowledge about N400 and LPC, time windows of 250–500 and 550–1200 ms were specified for the N400 and LPC components, respectively. These time windows were defined independent of the analysis of the MPH manipulation. In addition, due to the possible component overlap (Roehm et al. 2007; De Grauwe et al. 2010; Rommers et al. 2013) and the potential carryover effect of MPH between consecutive time windows, the N400 negativity was examined across two time windows, 1) the early N400 (250–350 ms) and 2) the late N400 (350–500 ms); the LPC was also examined across two time windows: 1) the early LPC (550–900 ms) and 2) the late LPC (900–1200 ms).

A whole-brain cluster-based permutation test was conducted in FieldTrip (1000 randomizations, *P* < 0.05 corrected for multiple comparisons across 24 electrodes: Fz, F3/4, F7/8, FCz, FC1/2, FC5/6, Cz, C3/4, CP1/2, CP5/6, Pz, P3/4, P7/8, and O1/2). The advantage of the permutation test is that it has a strict control of the multiple comparisons problem by computing significance probabilities using a nonparametric method (Maris and Oostenveld 2007; Oostenveld et al. 2011). However, this procedure only allows pairwise comparisons. Therefore, after confirming the replication of the N400-LPC effects in the placebo condition, we firstly focused on contrasting the ERP effects (i.e., N400, LPC, pre-N400) between MPH and placebo conditions, in the Semantic and the Font-size tasks separately. Second, these ERP effects were also compared between the two tasks to further verify whether the differences in MPH-induced task-dependent effects were valid. Third, to examine whether the order of drug administration modulated the MPH-induced effect (The cluster-based permutation tests did not support such multiway testing. Therefore, we conducted a classical quadrant analysis in repeated ANOVAs.), we conducted 2(MPH) × 2(Congruence) × 2(Anteriority) × 2(Hemisphere) × 2(Order) mixed factorial repeated ANOVAs, after averaging the mean voltage over four quadrants (left/right anterior, F7/8, F3/4, FC1/2, FC5/6; left/right posterior, CP1/2, CP5/6, P3/4, P7/8). Fourth, we have conducted an exploratory analysis on the 0–250 ms around the target word onset to examine if there were any MPH effect prior to the N400 time window. Last, we conducted another exploratory analysis relating the ERP effects to participants’ baseline performance and SEBR data to investigate if there were any modulation effects from their baseline performance.

## Results

### Cardiovascular and General Modulation Effects of MPH

The averaged values of the cardiovascular parameters are shown in Table 1. Results from the 2 MPH (MPH vs. Placebo) × 2 Time (before vs. after capsule administration) repeated measures ANOVAs showed that there were significant interactions of MPH × Time on participants’ heart rate (HR), systolic blood pressure (BP), and diastolic BP (*P*s < 0.003). Consistent with previous findings, further comparisons revealed that after capsule administration, all three parameters were higher in the MPH than in the placebo conditions (all *P*s < 0.012) (Ballard et al. 1976; Volkow et al. 2003; Cooper et al. 2005; Froböse et al. 2018; Baas et al. 2020), though they were very similar between the two conditions before capsule administration (all *P* > 0.56). Planned comparisons showed that in the placebo session, participants’ HR and systolic BP significantly decreased over the course of testing (*P*s < 0.001), though their diastolic BP did not change (*P* = 0.46), while in the MPH session, participants’ HR (*P* = 0.051) and systolic BP (*P* = 0.85) did not show any significant change; their diastolic BP increased over the course of testing (*P* < 0.001).

**Table 1:** Cardiovascular and general modulation effects of MPH

		Time	Placebo^a^	MPH	Difference
Cardiovascular^b^	Syst. BP	Before	116.2 (10.8)	115.5 (10.9)	ns
		After	112.6 (10)	115.7 (10.5)	*t*(41) = 2.63, *P* = 0.012^*^
	Diast. BP	Before	70.9 (7.5)	71.0 (7.5)	ns
		After	71.3 (6.7)	74.2 (6.7)	*t*(41) = 3.37, *P* = 0.002^*^
	HR	Before	68.1 (13.6)	67.9 (10.6)	ns
		After	61.0 (10.9)	65.8 (12.5)	*t*(41) = 3.62, *P* = 0.001^*^
Tasks	Number cancellation (ms)		3.52 (0.69)	3.50 (0.70)	ns
	Number cancellation (No.)^c^		0.21 (0.95)	0.24 (0.98)	ns
	Operation span (short)		26.0/30 (4.8)	26.0/30 (5.2)	ns
	Asymmetry span (short)		17.9/30 (4.5)	17.6/30 (4.5)	ns

Note: “Before” refers to before capsule administration; “After” refers to after capsule administration

^a^The value in the parentheses represents standard deviations. The group difference was tested with a planned paired sample *t*-test following significant interaction. For exploration purpose, the values reported here were not corrected for multiple comparisons

^b^The cardiovascular parameters were measured two times before and two times after the capsule administration. The average values of the “before” and the “after” measurements were reported in the table

^c^The number of cancellation was tested about 60 min after the capsule administration. The dependent variable is the number of the correctly crossed out numbers minus the incorrectly crossed out numbers. In this task, participants were required to cross out all the numbers 6 and 9 from 28 rows of 35 digits

^*^ *P* < 0.05

Regarding the VAS questionnaire, following Bond and Lader's (1974) method, we evaluated participants’ subjective feelings on three factors (i.e., alertness, contentedness, and calmness) extracted from their self-ratings on the 16 questions. Results from the 2 MPH (MPH vs. Placebo) × 2 Time (before vs. after capsule administration) repeated measures ANOVAs did not find any significant effects (all *P* > 0.11). With similar statistical methods, there was no significant change in participant’s self-reported physical symptoms, visual attention (as measured by the number cancellation test), or general WM capacities (as measured by the two shortened WM tasks) after taking MPH.

Together, the observation of cardiovascular effects after MPH administration demonstrated that our MPH manipulation was successful. The enhancement of autonomic arousal is related to the MPH effect on the catecholaminergic system. No participant had an aversive response to the drug. The lack of any MPH effect in the self-report questionnaires and WM tasks suggests that any MPH-induced change observed in participants’ sentence comprehension performance could not be simply attributed to the general modulation effects of MPH on cognition.

### Sentence Comprehension: Behavioral Results

**Figure 1:**
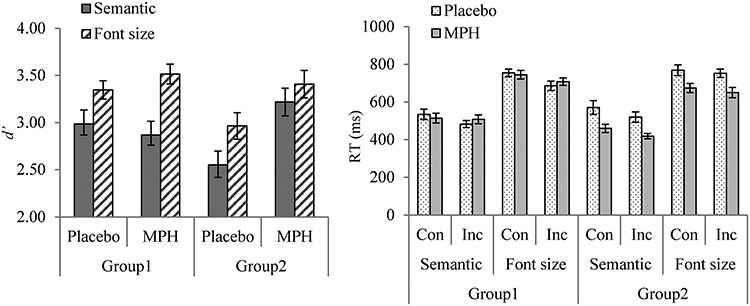
Mean response accuracy (*d′*) and RTs for each condition in Order 1 and Order 2 separately. Order 1 took the MPH-placebo order, while Order 2 took the reversed order. “Con” represents congruent condition and “Inc” represents incongruent condition. The error bars represent corrected standard error of the mean (Cousineau 2005). For illustration purposes, the figure uses raw RTs for subjects’ performance in each condition, despite the fact that all analyses were conducted using log-transformed RTs.

Accuracy and response times (RTs) to the comprehension questions were reported in Table 2. Participants were generally attentive and accurate during the experiment (mean accuracy = 94%, SD = 3%; mean RT = 608 ms, SD = 120 ms). Results from repeated measures ANOVAs are presented in Table 3 and Figure 1.

**Table 2:** Accuracy and RTs to the comprehension question in the sentence comprehension task

		Semantic judgment		Font-size judgment	
		Placebo	MPH	Placebo	MPH
Accuracy	Congruent	0.94	0.94	0.95	0.97
	Incongruent	0.89	0.92	0.92	0.93
RT (ms)	Congruent	551	486	761	711
	Incongruent	500	465	717	680

**Table 3:** Behavioral results of the sentence comprehension experiment

DVs	*d′*			RT (ms)		
	*F*	MSE	*P*	*F*	MSE	*P*
MPH	7.22	2.056	0.010^*^	7.44	201 448.68	0.009^*^
MPH × Order	7.64	2.175	0.009^*^	8.86	239 739.32	0.005^*^^*^
Task	4.09	2.864	0.050^*^	84.42	3 923 673.98	<0.001^*^^*^^*^
Task × Order	1.77	1.238	0.191	0.01	642.57	0.907
MPH × Task	0.00	0.000	0.971	0.11	665.61	0.746
MPH × Task × Order	4.13	0.883	0.049^*^	0.01	77.02	0.912
Order	0.18	0.153	0.675	0.09	18 454.35	0.772
Congruence	n.a.			18.03	114 096.57	<0.001^*^^*^^*^
Congruence × Order	n.a.			0.19	1213.35	0.664
MPH × Congruence	n.a.			4.22	7967.12	0.047^*^
MPH × Congruence × Order	n.a.			3.92	7392.89	0.055
Task × Congruence	n.a.			0.00	11.45	0.973
Task × Congruence × Order	n.a*.*			1.28	12 482.26	0.265
MPH × Task × Congruence	n.a.			0.47	1119.43	0.499
MPH × Congruence × Task × Order	n.a.			0.01	20.64	0.927

*Note*: n.a., not available

^*^ *P* < 0.05

^**^ *P* < 0.01

^***^ *P* < 0.001

#### Accuracy (*d′*) Analysis

As shown in Table 3, there were significant main effects of MPH and task at group level. Participants were more accurate in the MPH condition than in the placebo condition (3.27 vs. 3.04) and more accurate in the Font-size task than in the Semantic task (3.39 vs. 3.02). Importantly, there was an interaction of MPH × Task × Order. Planned analyses showed that the main effect of MPH was only significant when participants took the MPH in session 2 (Order 2), in which participants were better at detecting both semantic and perceptual incongruence while they were on MPH (3.35 vs. 2.90, *F*(1, 19) = 14.29, MSE = 4.04, *P* = 0.001). On the other hand, when participants took the MPH in session 1 (Order 1), there was a main effect of task. Participants were generally more accurate at detecting perceptual than semantic incongruence (3.40 vs. 2.97, *F*(1, 19) = 7.45, MSE = 4.13, *P* = 0.013). Although we tried to match the task difficulty between the two tasks through a pilot study, the observation of an advantage of detecting perceptual incongruence in a larger sample was not surprising. Perceptual processing in our current experiment was very clear-cut and involved a finite set of visual features, while determining semantic fit was more complex due to the possibility of degrees of fit (Hagoort 2003). The absence of an MPH effect in the participants taking Order 1 (i.e., taking MPH in session 1) suggested that there might be a practice effect, which boosted Order 1 participants’ performance in session 2 when they took placebo. To test this hypothesis, we examined the Task × MPH interaction in each session separately, with MPH as a between-group variable. However, the MPH effect was not significant in either session (session 1, *F*(1, 40) = 2.71, MSE = 1.66, *P* = 0.107; session 2 *F*(1, 40) = 1.03, MSE = 0.54, *P* = 0.32) (In session 1 only, participants also showed more sensitivity to the perceptual than the semantic incongruence (semantic, 2.84 vs. font size, 3.24; *F*(1, 40) = 7.32, MSE = 3.46, *P* = 0.010). The interaction of Task × MPH was not significant.).

#### RT Analysis

As shown in the right panel of Figure 1, there was a main effect of MPH: participants responded faster in the MPH relative to the placebo condition (583 vs. 633 ms). This is consistent with the finding that MPH speeds up response time (Elliott et al. 1997). In addition, there was a main effect of task, with participants responding faster when they were required to judge semantic congruency than font-size difference (501 vs. 717 ms). This RT difference was expected as the semantic violations could be detected during online reading, and participants were ready for the button press even before the prompt in the Semantic task. However, they had to wait for and process the probe word in the Font-size judgment task. Additionally, there was a main effect of congruency, reflecting that participants were faster in judging incongruent than congruent conditions in both tasks. This congruency effect suggested that participants needed longer time to confirm that a sentence was congruent than to respond to the presence of an incongruency (Hagoort 2003). Furthermore, there were interactions of MPH × Congruence and MPH × Order. Further analysis showed that after taking MPH, participants responded faster to the question following the congruent condition (i.e., semantic congruent condition in the Semantic task and same font size in the Font-size task), *t*(41) = −2.75, *P* = 0.009, while there was only an marginal improvement in the incongruent condition, *t*(41) = −1.84, *P* = 0.073. The fact that participants showed larger improvement in the congruent condition might be because that they already responded very fast in the incongruent condition and there was a floor effect. Regarding the interaction of MPH × Order, when participants took placebo first (Order 2), they were overall faster on MPH than placebo (placebo, 653 ms vs. MPH, 550 ms, *t*(21) = −4.32, *P* < 0.001), whereas there was no MPH effect when they took MPH first (placebo, 614 ms vs. MPH, 618 ms, *t*(21) = −4.32, *P* = 0.869). We further examined the MPH × Order interaction within each session with MPH as a between-group variable. The main effect of MPH was not significant in either session (*P*s > 0.23).

#### Summary of Behavioral Results

Together, the results from both accuracy and RT analyses suggested that MPH enhanced participants’ performance in detecting perceptual and semantic incongruence. However, the observed MPH effect in these behavioral data interacted with the order of drug administration. Participants who took the placebo first and MPH second showed greater enhancements. This might be the result of an interaction of the MPH effect and a practice effect. It should be noted that although there was a task difference in both accuracy and RT data, we did not observe a differential effect of MPH on the Semantic and the Font-size task, as the interactions of MPH × Task were not significant.

### Sentence Comprehension: Replication of N400 and LPC Effects in the Placebo Condition

**Figure 2:**
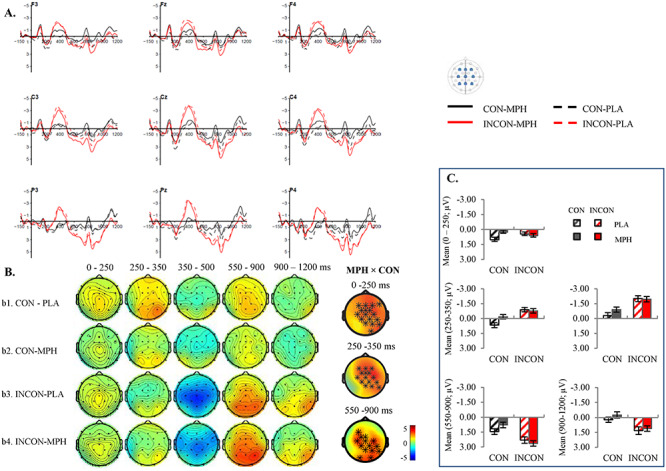
Grand-averaged ERPs (*n* = 42) in the Semantic task. (*A*) Waveforms at nine representative electrodes timed-locked to the critical nouns in the semantically congruent (CON) versus incongruent (INCON) and MPH versus placebo (PLA) conditions. The negativity is plotted upward. For illustrative purpose only, a 15-Hz low-pass filter has been applied on the waveforms. (*B*) Scalp distributions of the semantically congruent and incongruent conditions on MPH and placebo. The electrodes that were included in the significant cluster of MPH × Congruence interaction were plotted as well. The positive interactions suggested that the mean amplitude difference between congruent and incongruent (INC–CON) conditions was smaller in the MPH than the placebo condition, and the negative interaction suggested an opposite direction (^*^P < 0.01, ×*P* < 0.05). (*C*) Mean amplitudes in the electrodes that showed significant interaction effect in each time window. Consistent with ERPs in (*A*), the negativity is plotted upward as well. For the time windows that showed no significant interactions (i.e., 350–500 and 900–1200 ms), we plotted the mean amplitudes for the same electrodes as in the earlier time window (i.e., 250–350 and 550–900 ms, respectively), in order to show the continuation of the previous effect. The error bars represent corrected standard error of the mean for a within-subject design. As discussed in the Methods section, the observation of significant clusters did not provide information on the exact spatial extent.

**Figure 3:**
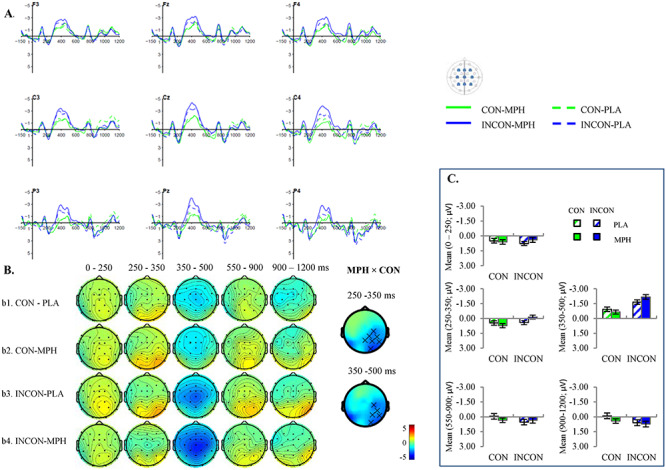
Grand-averaged ERPs (*n* = 42) in the Font-size task. (*A*) Waveforms at nine representative electrodes timed-locked to the critical nouns in the semantically congruent (CON) versus incongruent (INCON) and MPH versus placebo (PLA) conditions. The negativity is plotted upward. For illustrative purpose only, a 15-Hz low-pass filter has been applied on the waveforms. (*B*) Scalp distributions of the semantically congruent and incongruent conditions on MPH and on placebo. The electrodes that were included in the significant cluster of MPH × Congruence interaction were plotted as well. The negative interaction suggested that the mean amplitude difference between congruent and incongruent (INC–CON) conditions was larger in the MPH than the placebo condition (^*^P < 0.01, ×*P* < 0.05). (*C*) Mean amplitudes in the electrodes that showed significant interaction effect in each time window. Consistent with ERPs in (*A*), the negativity is plotted upward as well. For the time windows that showed no significant effect (i.e., 100–150, 550–900, and 900–1200 ms) in the Font-size task, we plotted the mean amplitudes for the same electrodes in each window as in the Semantic task, in order to allow cross-task comparisons. The error bars represent corrected standard error of the mean for a within-subject design.

The grand-averaged ERPs from representative electrodes in the Semantic and the Font-size tasks are presented in Figures 2 and 3. Figures with results at all electrodes are available in the Supplementary Material A. The order of drug administration did not significantly modulate participants’ EEG response (see discussion in the Supplementary Material B). Therefore, we collapsed over the Order 1 and Order 2 groups.

As shown in the figures, the critical noun elicited the pattern characteristic of ERPs to visually presented verbal stimuli, including an N1-P2 complex in the first 200 ms after word onset, followed by an N400 component, and an LPC in the Semantic task only. After taking the placebo, in the Semantic task (Fig. 2), the semantically incongruent condition elicited a more negative N400 response that was widely distributed between 250 and 500 ms (*P* = 0.002; early time window, *P* = 0.002; late time window, *P* = 0.002). This N400 effect was followed by an LPC between 550 and 1200 ms with a typical posterior distribution (*P* = 0.032; early time window, *P* = 0.022; late time window, *P* = 0.016). In the Font-size task, an N400 effect was only observed in the late N400 time window (*P* = 0.028) with a centro-parietal distribution. There was no semantic congruency effect in either the early N400 or any LPC time windows. This result was consistent with the previous finding that LPC only occurred in task-relevant or attentional conditions, in which participants were explicitly required to focus on semantic processing (Holcomb 1988; Gevins et al. 1997; Kuperberg 2007). Moreover, a direct comparison between the Semantic and the Font-size task in the late N400 time window showed that the magnitude of the N400 effect was larger in the Semantic task (*P* = 0.006), which was a result of a less negative N400 amplitude to the semantically congruent sentences in the Semantic task (*P* = 0.002), while there was no significant difference for the semantically incongruent sentences.

In summary, these results replicated the classical N400-LPC effect, which was observed when participants were instructed to do purposeful semantic processing (i.e., Semantic task). In line with many previous findings, we found that participants still processed semantic information even when they were instructed to focus on other aspects of the stimuli (e.g., font size). However, they processed semantic information to a shallower degree as indexed by a relatively delayed and smaller N400 effect in the Font-size task (Holcomb 1988; Brown and Hagoort 1993; Chwilla et al. 1995; Brown et al. 2000). In the following sections, we examine the modulation effects of MPH in the Semantic and the Font-size tasks separately.

### Modulation Effects of MPH in the Semantic Task

We examined the modulation effects of MPH in the Semantic and the Font-size tasks separately with a full factorial design (i.e., MPH × Congruence).

In the Semantic task (see Fig. 2), when participants were instructed to do purposeful semantic processing, there was a main effect of semantic congruency. Participants generally showed a more negative N400 response in the semantically incongruent compared to the congruent condition (*P* = 0.002). There was no main effect of MPH. Importantly, there was a significant interaction of MPH × Congruence in the early N400 time window over the centro-parietal regions (*P* = 0.004). Participants showed a reduced N400 effect on MPH compared to placebo. Further analyses on the interaction revealed that the smaller N400 effect induced by MPH was caused by a smaller reduction of N400 response in the congruent condition in the MPH condition (*P* = 0.03), while there was no significant change in the incongruent condition (*P* = 0.40). Moreover, MPH had an influence on the later stage of sentence processing as demonstrated by an interaction of MPH × Congruence in the early LPC time window (550–900 ms; *P* = 0.008). As depicted in Figure 2*C*, participants showed a larger LPC effect in the MPH compared to the placebo conditions. No further comparisons reached significance.

### Modulation Effects of MPH in the Font-size Task

In the Font-size task, during which participants were instructed to focus on processing the perceptual aspects of the sentences, participants still automatically processed semantic information as demonstrated by a significant main effect of the semantic congruency (*P* = 0.002). There was no main effect of MPH in any predefined time window. Importantly, there was a significant interaction of MPH × Congruence in both the early and the late N400 time window (*P*s < 0.05), and thus we collapsed across the two time windows. The interaction was significant in the entire N400 time window (*P* = 0.024). Participants showed a larger N400 effect over the right centro-parietal electrodes on MPH than placebo. Interestingly, in contrast to the Semantic task, planned comparison showed that the MPH × Congruence interaction was driven by a more negative N400 amplitude in the incongruent condition when participants received MPH compared to a placebo (*P* = 0.008). There was no such MPH-induced difference in the congruent condition (*P* = 0.19). In addition, there was no MPH × Congruence interaction in the LPC time windows.

### Comparison Between the Semantic and the Font-size Tasks

**Figure 4:**
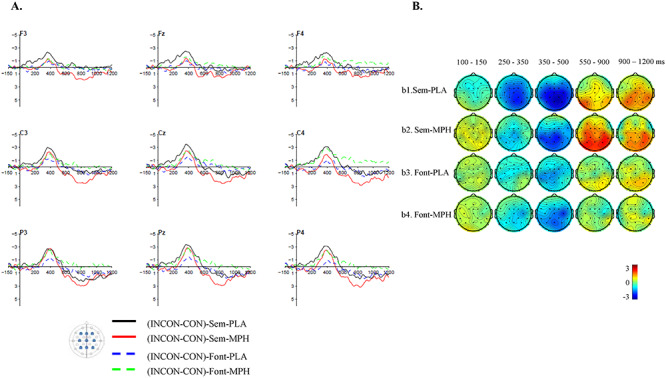
Grand-averaged amplitude differences between the semantically congruent (CON) and incongruent (INCON) sentences in the MPH and placebo (PLA) conditions in the Semantic (Sem) and Font-size (Font) tasks. The negativity is plotted upward.

Results from the above analyses suggested that MPH had different effects on semantic processing in the Semantic and the Font-size tasks. To determine whether MPH indeed had a task-dependent effect on semantic processing, we conducted direct comparisons between the ERP’s effects in the two tasks (i.e., [MPH_incongruent_- MPH_congruent_]—[Placebo_incongruent_- Placebo_congruent_]). In all predefined time windows except the [900–1200 ms] (*P* = 0.196), there were significant differences between the MPH-induced changes in the ERP effects between the two tasks (all *P* < 0.042). These results confirmed that the effect of MPH differed by tasks. To better visualize the differences between the ERP effects in the Semantic and the Font-size tasks, the grand-averaged amplitude difference between congruent and incongruent sentences in the four conditions (MPH × Task) are presented in Figure 4.

Overall, we found a task-dependent effect of MPH on semantic processing during sentence comprehension. When semantic processing was task-irrelevant, participants showed higher sensitivity to the semantic incongruency after receiving MPH than a placebo, as evidenced by a larger N400 effect. In contrast, comparing to placebo, when semantic processing was task-relevant, participants showed a smaller reduction in the N400 amplitude when the critical word was semantically congruent. In addition, MPH induced an elevated LPC effect in the Semantic task indicating a more elaborate and extended semantic processing. Last, for exploratory purposes, we also examined whether there was any correlational effect between the MPH-induced changes in the behavioral data and the EEG response (see Supplementary Material C). None of the correlations was significant, even without correcting for the multiple comparisons. Given that our sample size is not sufficient for a strict correlational test and the underlying mechanisms supporting the online and offline semantic processing differed in many aspects, further studies are needed to examine the relation between the MPH effects on the behavioral and the EEG data.

### Exploratory Analysis: The Early Effect of MPH

We have conducted an exploratory analysis on the pre-N400 brain responses. Traditionally, EEG components within 200 ms (e.g., N1, P2) after stimulus onset are most commonly associated with automatic perceptual processing (Lijffijt et al. 2009) or early syntactic processing (e.g., lexical selection, word category processing) during sentence comprehension (Hahne and Jescheniak 2001; Van den Brink and Hagoort 2004; Bornkessel-Schlesewsky and Schlesewsky 2019) and are not commonly discussed in the N400 studies. However, recently, some researchers suggested that certain early effects reflect the prediction of a specific word form during language processing (Brothers et al. 2015; Hagoort 2017; Nieuwland 2019; Pickering and Gambi 2018). For exploratory purposes, we examined a 0–250 time window around the target word onset to see if MPH has any influence on the early EEG components in the pre-N400 time window. It should be noted that because the relation between the pre-N400 ERPs and language processing is not clear yet, we treated this analysis as an exploratory one.

The results showed that the interaction of MPH × Congruence was already evident in this time window in the Semantic task (*P* = 0.011) but was not in the Font-size task. Follow-up tests revealed that the MPH × Congruence interaction in the Semantic task was driven by a more negative response in the congruent condition over the frontal–central electrodes after receiving MPH compared to placebo (*P* = 0.002), while there was no significant change in the incongruent condition. Based on visual inspection of the grand-averaged waveform as shown in Figure 2, this early effect seemed to be most robust between 100 and 150 ms. For exploratory purposes, we conducted a step-wise analysis in which we tested for the effect in 10 ms time windows between 80 and 160 ms with a FDR control for multiple comparisons to further examine the timing of this effect (see Supplementary Material D). The corrected results showed that this effect was significant between 130 and 150 ms. In addition, this interaction had a similar distribution as the interaction observed in the early N400 time window, which was most pronounced over right centro-parietal electrodes. As a result, we could not rule out the possibility that the MPH-induced reduction on the N400 effect was a carryover effect from this pre-N400 difference. This possibility was partially supported by the finding that when we used a poststimulus baseline of [0–100 ms] to control for the early differences between conditions, the interaction of MPH × Congruence disappeared in all later time windows (see Supplementary Material E) (It should be noted that there were no significant effects in any time windows on the adjective prior to the critical noun (see Supplementary Material F for the results). We examined ERPs using longer prestimulus baselines (e.g., [−300 to 0 ms], [−200 to 0 ms]) to further control for the potentially overall MPH effects on the EEG signals. However, none of those analysis produced a significantly different pattern than the one reported in the paper with a classical [−150 to 0 ms] baseline.). Based on the results from previous studies, we suggested that MPH might enhance participant’s processing of contextually congruent words at an early stage. However, this analysis was exploratory and requires replication in future studies.

### Exploratory Investigation of the Relationship Between MPH-Induced Effects on Semantic Processing and Participants’ Base Levels of Performance

Some previous studies have suggested that the cognitive effects of MPH are modulated by individuals’ base levels of performance (Mehta et al. 2000; Frank et al. 2007; Cools and D'Esposito 2011; Van der Schaaf et al. 2013). Therefore, we conducted an exploratory analysis on the relationship between the MPH-induced ERP effects and individual’s base levels of performance with Pearson Product–Moment Correlation tests. The detailed analyses are presented in Supplementary Material G. There are several interesting findings. First, the results showed that individuals with better WM capacity showed smaller changes in the N400 effect size when they were on MPH than on placebo (*P* = 0.006). Second, although the changes in participants’ cardiovascular parameters demonstrated that our MPH manipulation was effective, we did not observe any significant changes in participants’ spontaneous eye blink rate after receiving MPH. This result challenges the claim that SEBR is a good clinical predictor for dopaminergic activity.

## Discussion

This is the first study demonstrating a clear neuropharmacological effect of MPH on semantic processing during sentence comprehension in a healthy population. Results from the current study confirmed that semantic incongruency always elicits an N400 effect irrespective of task requirements or drug administration, which demonstrated the automaticity of semantic processing (Holcomb 1988; Deacon and Shelley-Tremblay 2000; Küper and Heil 2009). The main novelty of the current finding is that MPH affects language processing in a task-dependent manner: MPH “attenuated” the N400 effect when semantic processing was task-relevant but “elevated” the N400 effect when semantic processing was task-irrelevant. Further analyses revealed that the MPH-induced attenuation of the N400 in the task-relevant condition was caused by a more negative N400 amplitude in the semantic congruent condition than on placebo, while the larger N400 effect in the task-irrelevant condition was caused by a more negative N400 amplitude in the semantically incongruent condition than on placebo. In addition, in the task-relevant condition only, the attenuated N400 was followed by an increased LPC effect and possibly preceded by an attenuated early negativity. Overall, our results demonstrate a causal link between catecholaminergic activities and semantic processing. We suggest that catecholamine exerts its impact on language through mediating effects of the projections between the striatum and the PFC, amplifying the salience of semantic information during language processing. The increased extracellular catecholamine levels in the striatum supported semantic combinatorial processing within the PFC. Our findings have a number of theoretical implications for neurocomputational models of language processing.

### Task-Dependent Effect of Catecholaminergic Drug Administration

The different effects of MPH on semantic processing in the Semantic and the Font-size tasks resonate with previous findings that MPH has a task-dependent influence on cognitive functions (Volkow et al. 2004; Durstewitz and Seamans 2008; Ter Huurne et al. 2015). However, in contrast to the account that MPH generally enhances participants’ focus on task-relevant information while inhibiting the processing of task-irrelevant information, our results showed that MPH prompted semantic processing even when language processing per se was task-irrelevant. These results are most consistent with the claim that the higher CA level amplifies the saliency of crucial information, such as meaning (Hagoort 2017, 2018, 2019), even if it is task-irrelevant or nonrewarding (Volkow et al. 2001, 2004; Bromberg-Martin et al. 2010; Ter Huurne et al. 2015). In a recent study, Ter Huurne et al. (2015) have demonstrated that although MPH generally improved participants’ accuracy of identifying the gender of target face stimuli, it impeded face processing as indexed by longer reaction times when the distractors were also faces compared to scrambled stimuli. Ter Huurne and colleagues concluded that MPH amplifies the saliency of objects from the target category, irrespective of whether processing of these objects was task-relevant or not. Most recently, Westbrook et al. (2020) have shown that MPH could promote individuals’ willingness to exert cognitive effort by altering the effects of benefits versus costs (Westbrook et al. 2020). In line with these findings, the increased N400 effect in the Font-size task suggests that the intrinsic relevance of language for communication makes its processing mandatory. A higher CA level further amplified the saliency. As a result, participants exerted more cognitive effort even when semantic processing was orthogonal to the processing goal. It is noteworthy that the observed interaction of MPH and semantic processing could not be fully explained by the general effects of increased CA levels on improving attention, as there was no significant change on participants’ general processing speed or WM capacity.

With respect to the attenuated N400 effect in the Semantic task, the planned comparisons showed that the reduced N400 effects were caused by a more negative N400 amplitude in the semantically congruent condition, whereas there was no MPH effect in the incongruent condition. Traditionally, a robust and consistent finding is that the size of the N400 effect is proportional to the difficulty of semantic processing. The N400 amplitude is increased as a result of a lower predictability of the target word or a poor fit between the target word and the semantic context (Kutas and Federmeier 2011; Bornkessel-Schlesewsky and Schlesewsky 2019). Therefore, the smaller reduction of the N400 response in the MPH condition suggests that participants did not respond to the degree of semantic fit or predictability of upcoming words in the same way as they did in the placebo condition (Bornkessel-Schlesewsky and Schlesewsky 2019). The smaller reduction might be caused by the fact that when participants were on MPH, they did not reduce the processing effort even when they detected the semantic fit between the upcoming word and the context. We believe that the Semantic task induced an external saliency-driven response to the language input. In this context, semantic incongruities were already maximally salient and not further boosted by a saliency-related signal induced by MPH via its influence on the DA system. The expected congruent semantic information, on the other hand, still profited from an MPH-induced saliency boost, since due to the expectedness of the target word, its saliency was not increased by a language system internal prediction. In the Font-size task, the opposite situation was at stake, since the task requirements in this task did not induce an externally driven saliency response to semantically unexpected items. Hence, the MPH induced a saliency boost to the prediction error that was task-irrelevant, resulting in an increased N400 amplitude for the incongruent condition.

Regarding the larger LPC effect in the Semantic task after receiving MPH compared to placebo, we suggest that a higher CA level enhances general conflict monitoring or repairing processes required during elaborate language processing (Münte et al. 1998; Van Herten et al. 2006; Kos et al. 2012; Leckey and Federmeier 2020). The observation of an elevated LPC effect in the current study suggests that participants might exert more effort to revise or repair the semantically incongruent sentences after receiving MPH.

Last, one of the important findings in the current experiment is the MPH-induced semantic effect in the pre-N400 time window, which was less expected. Most of the previous psycholinguistics studies examining N400 effects did not observe or report such pre-N400 negativities, and many previous pharmacological-EEG studies did not observe a significant MPH effect on early attentional components such as the N1/P2 (Hink et al. 1978; Anderer et al. 2002; Cooper et al. 2005; Hermens et al. 2007; Studer et al. 2010; Dockree et al. 2017). Importantly, it should be noted that this early MPH effect was only evident in the Semantic task but not in the Font-size task in our current experiment. The similar pattern of the MPH × Congruency interaction in the pre-N400 and the N400 time windows left open the question whether the MPH × N400 effect interaction was a carryover effect from this early time window. Regarding this very early negativity itself, several studies have demonstrated that an orthographic or phonological overlap or a strong lexical–semantic prediction between the predicted target and the actual input could lead to a reduced early negativity between 100 and 250 ms (Van den Brink and Hagoort 2004; Grainger and Holcomb 2009; Dikker and Pylkkanen 2011; Lau et al. 2013; Brothers et al. 2015). Therefore, we suggested that MPH might amplify the saliency value of word processing at a very early stage. This claim was supported by the results from our exploratory analysis that only sentences with relative high semantic constraints elicit an MPH × Congruency interaction in the N100–150 time window, while sentences with relatively low semantic constraints only elicit an MPH × Congruency interaction in the N400 time window (see Supplementary Material H). Moreover, given some previous findings that there was a dissociation between the pre-N400 negativity and the N400 (Connolly and Phillips 1994; Van den Brink and Hagoort 2004; Lau et al. 2013), we suggest that the observed MPH × N400 interaction is unlikely to be merely a carryover effect from the 100 to 150 ms time window, since slight differences in topography suggest at least partially nonoverlapping neuronal contributions.

### Implications for the Influence of Catecholaminergic Mechanism on Language Processing

**Figure 5:**
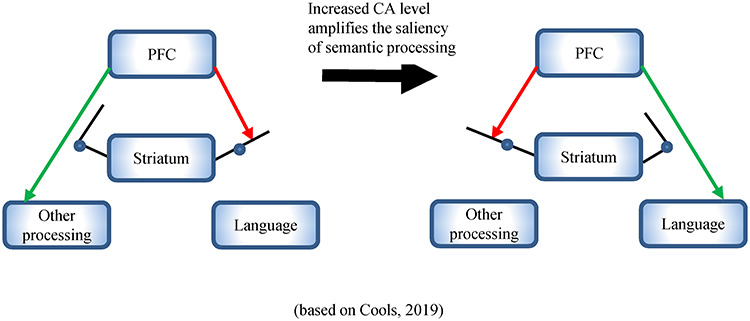
Hypothesized neurotransmitter mechanisms of the catecholaminergic system’s gating function. The figure is adapted from Figure 4 in Cools (2019). We hypothesized that the CAergic system could up- and downregulate language processing by modifying the striatum to prefrontal cortex (PFC) projections. An increased striatum CA level provides a relevance signal for the PFC to amplify the saliency of language input, and thus supports the semantic combinatorial processing within the PFC.

The most important implication of the current study is that it reveals a causal role of the catecholaminergic system in language processing. Previous pharmacological studies have demonstrated that the effect of MPH on cognition is most commonly attributed to altered catecholamine availability in the striatum and the PFC, which contain a large number of CA receptors (see Cools and D’Esposito 2011, for a review). More specifically, some studies have suggested that the change of DA levels in the striatum might be a precondition for the observed effects at the PFC, as increased striatal DA levels regulate the cortical dynamics of PFC and thus influence its top–down control on cognitive functions (Clatworthy et al. 2009; van Schouwenburg et al. 2010; Fallon et al. 2017). Results from our current study further corroborated the involvement of the striatum and the PFC in language processing. Specifically, the task-dependent characteristic of the MPH effects suggests that the CA level may influence language processing through the projections between the striatum and the PFC regions, whereby the saliency of language-relevant information can be increased. In the context of psycholinguistic research, many previous studies have focused on the role of the PFC, which has been shown to support complex sentence processing through maintaining and updating semantic interpretations (see Hagoort 2017, for a review). However, recent neuroimaging studies have demonstrated that the striatum also plays a crucial role in cognitive functions, such as WM, cognitive control, action planning, and language processing (Gruber et al. 2006; Hazy et al. 2007; Durstewitz and Seamans 2008; Snijders et al. 2010). The striatum provides a dynamic gating mechanism through the cortico-striatal loop functions and thus could momentarily inhibit or enhance certain cortical functions. For example, Snijders et al. (2010) found that during language processing, the striatum was functionally connected to low-level visual regions for processing ambiguous words while connected to the high-level regions such as frontal and temporal cortex for processing ambiguous sentences. As illustrated in Figure 5, we suggest that increased striatum CA levels may provide a relevance signal for the PFC to amplify the salience of language-relevant information and thus support semantic combinatorial processing within the PFC, even when such processing is task-irrelevant. However, it should be noted that the precise nature of the CA influence on language processing remains a question for further research. For example, it was difficult to segregate whether DA or NA contribute more to the effects or whether CA influences language processing in a direct or indirect way. This is because the pharmacological and psychotropic nature of MPH is still unclear. It requires employing neuroimaging techniques such as in vivo PET to determine the pharmacological specificity of the MPH effects in future studies.

Additionally, although our results demonstrated an impact of MPH on semantic processing, we did not obtain any MPH-induced effects on participants’ WM capacity or general processing speed. This is consistent with the finding from a number of studies that there was no overall enhancing effect of MPH on cognition (Smith and Farah 2011; Paton et al. 2014). However, it should be noted that the relatively low dose of MPH used in the current study might constrain our ability to detect MPH effects on these tasks. Nonetheless, an interesting finding was that individuals with higher WM capacity were less susceptible to the effects of MPH administration, as they showed a smaller reduction in the N400 effect in the task-relevant condition. This finding supports the claim that the precise effect of CA agonists is modulated by individuals’ baseline WM capacity, which might be an index of their baseline CA levels (Kimberg et al. 1997; Mehta et al. 2000; Cools and D'Esposito 2011; Agay et al. 2014).

Lastly, our results pose a challenge to the long-held assumption that SEBR is a good predictor of participants’ dopaminergic activity by virtue of indexing DA receptor availability. This assumption was mainly based on the clinical observations that patients with a DA depletion problem always show a reduced SEBR (Taylor et al. 1999; Groman et al. 2014), while patients with an abnormally high DA level always show an elevated SEBR (Karson 1983; Nestor et al. 1997; Ohta et al. 1999; Kumar and Debruille 2004; Kiang et al. 2008; Ryu et al. 2012). However, if SEBR were a reliable predictor for an individual’s DA level, we would expect our healthy participants to generally show a higher SEBR after taking MPH, and the base level SEBR as measured in the placebo condition should modulate the MPH effects on their cognitive functions. Nevertheless, we obtained neither an MPH effect on participants’ SEBR nor a modulation effect of SEBR on any MPH × ERP interactions. Our results are more in line with the finding from animal models that the relation between SEBR and individual’s dopaminergic activity was not a straightforward one and maybe only direct DA agonists (MPH is an indirect agonist) could elevate SEBR (Kleven and Koek 1996).

### Implications for Models of Semantic Processing

Our results also have some implications for the neural mechanisms underlying semantic processing. The occurrence of an N400 effect in the task-irrelevant condition suggests that a full attentional control is not necessary for generating an N400 effect. Hence, semantic combinatorial processing is automatic and mandatory to some extent. On the other hand, a larger N400 effect in the task-relevant than irrelevant condition also suggests that N400 does not reflect a purely automatic process. In addition, the lack of an LPC response in the Font-size task confirmed that such later ERP component reflects a controlled process, which is affected by the processing goal (Kos et al. 2012). Together, all these results supported the argument that attentional control is involved in both N400 and LPC responses to some extent (Fiebach et al. 2001; Federmeier et al. 2002; Otten and Van Berkum 2009). The fact that MPH only influences the early time windows of both N400 and LPC effects is consistent with the argument that these ERPs might not be a unitary component (Baggio and Hagoort 2011).

### Limitations and Future Directions

It is important to consider that the present study had some limitations. First, as mentioned above, although our results clearly support a causal role of CA in semantic processing, the current results could not straightforwardly answer the question whether increased CA levels have a direct or an indirect effect on language processing. It is possible that MPH modulates language processing only through influencing other cognitive processes such as inhibition or WM. The answer to this question is important for understanding the nature of the influence of CA level on language processing. Second, although our results have shown that there was a general effect of MPH at the group level, future work needs to be done to carefully investigate the catecholaminergic modulation on language comprehension at the individual level. Previous studies have strongly suggested an inverted-U-shaped function between individuals’ CA level and their cognitive performance (Dodds et al. 2008; Cools and D'Esposito 2011). It is possible that some participants’ CA level was boosted to the level beyond their optimum and MPH administration actually impairs their performance.

Third, one might argue that the task-dependent effect of MPH occurred because the Font-size task is easier as indexed by a higher *d′* value. However, we believe that it is unlikely that the task-dependent effect of MPH is only or mainly driven by the task difficulty. Both the results from our current study and from previous studies (Oei and King 1980; Bullmore et al. 2003) demonstrated that the CAergic system has an influence on individual’s performance regardless of task difficulty. Most importantly, the MPH-induced effects on the online EEG were observed on the N400 in both the Semantic and the Font-size tasks. The N400 effect is a well-established EEG response related to semantic processing but not task difficulty (see Kutas and Federmeier 2011, for a review). Moreover, the task manipulation was introduced mainly to induce a difference in the saliency of the linguistic input, while the core language processing operations (i.e., lexical retrieval, syntactic and semantic analysis) remained the same. Hence, the offline tasks were not designed to equate the moments in time when task-relevant information became available or the moments in time at which a response could be given. Therefore, we think that the observed MPH effect is unlikely to be merely caused by task difficulty.

Last, for future investigations it would be important to assess the effect of MPH on other aspects of language processing, such as syntactic and pragmatic processing, which have been suggested to be impaired in patients with DA-related dysfunctions (Grossman et al. 2001; Friederici et al. 2003; Longworth et al. 2005; McNamara and Durso 2018). In addition, given the crucial role that the dopaminergic system plays in the regulation of reward mechanisms (Cools and D'Esposito 2011), it would be relevant to investigate whether the MPH effect on language processing is mediated by individuals’ motivation levels.

## Conclusion

Our current study demonstrates that MPH has a task-dependent enhancing effect on semantic processing. On the one hand, even when semantic processing per se is task-irrelevant, MPH amplifies the saliency of the linguistic input and enhances the neurophysiological response in situations where combining word meanings into a coherent interpretation of the utterance gets harder. On the other hand, when purposeful semantic processing is task-relevant, MPH influences the processing of semantically congruent sentences and enhances later revision processes. These results suggest that MPH’s enhancing effect on semantic processing may be carried out through the striatum to prefrontal cortex projections. As our capacity for language is a core system deeply rooted in our biological makeup and of great evolutionary importance, the increased striatal CA levels facilitate the involvement of the PFC and may be other brain regions in language processing through the cortico-striatal loop functions. Moreover, our exploratory analyses revealed that the MPH effect on purposeful semantic processing is modulated by individuals’ WM capacities. Participants with lower WM capacity showed a greater enhancement in language processing after receiving MPH. Taken together, we suggest that MPH enhances semantic processing by modulating the projections between the striatum and the PFC, which contains a large number of CA receptors and which is actively involved in language processing. Increased CA levels affect language processing by up- and downregulating the activity of the catecholaminergically innervated PFC and striatal regions.

## Supplementary Material

Supplementary_materials_bhaa204

## Notes

We would like to thank Roshan Cools, Sabine Kooijman, Zheng Ye, and Nathalie Boot for their discussion and help. *Conflict of Interest*: The authors declare no competing interests.
