# Supplementary material for: Catecholaminergic Modulation of Semantic Processing in Sentence Comprehension
Source: Cereb Cortex. 2020 Aug 8;30(12):6426–43. doi: 10.1093/cercor/bhaa204 (PMC7609945; doi:10.1093/cercor/bhaa204)
Supplement: Supplementary_materials_bhaa204 [file supplementary_materials_bhaa204.docx]

Supplementary materials

# Grand-averaged ERPs at all electrodes

#### a1. *Semantic* task


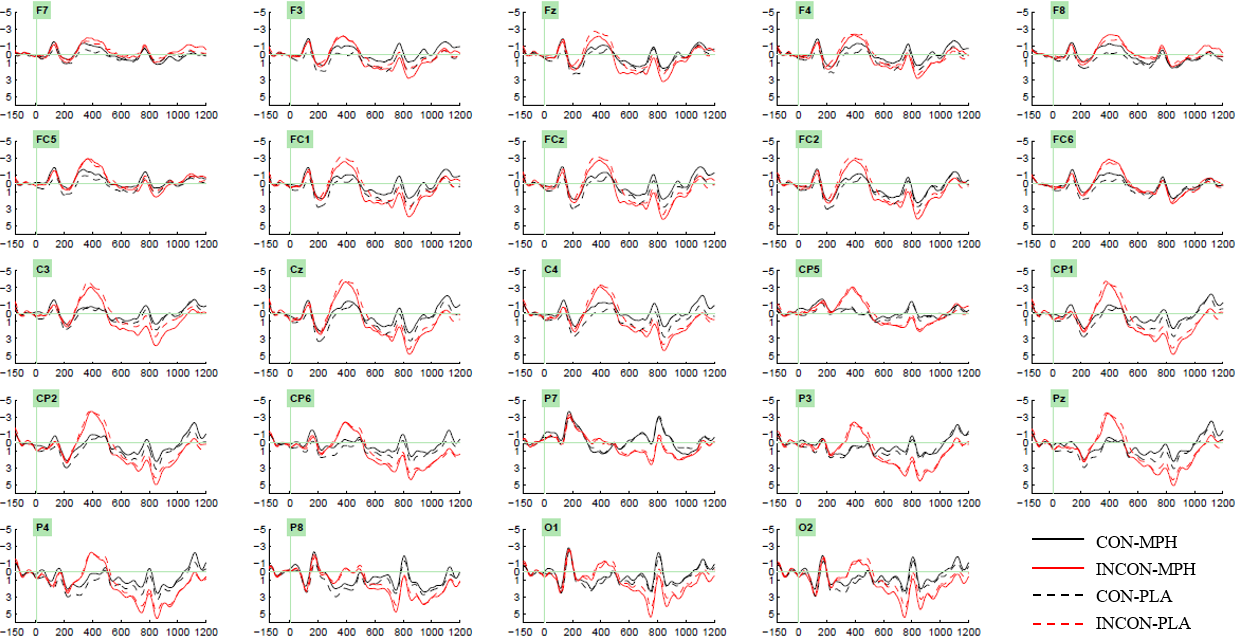


#### a2. Font-size task


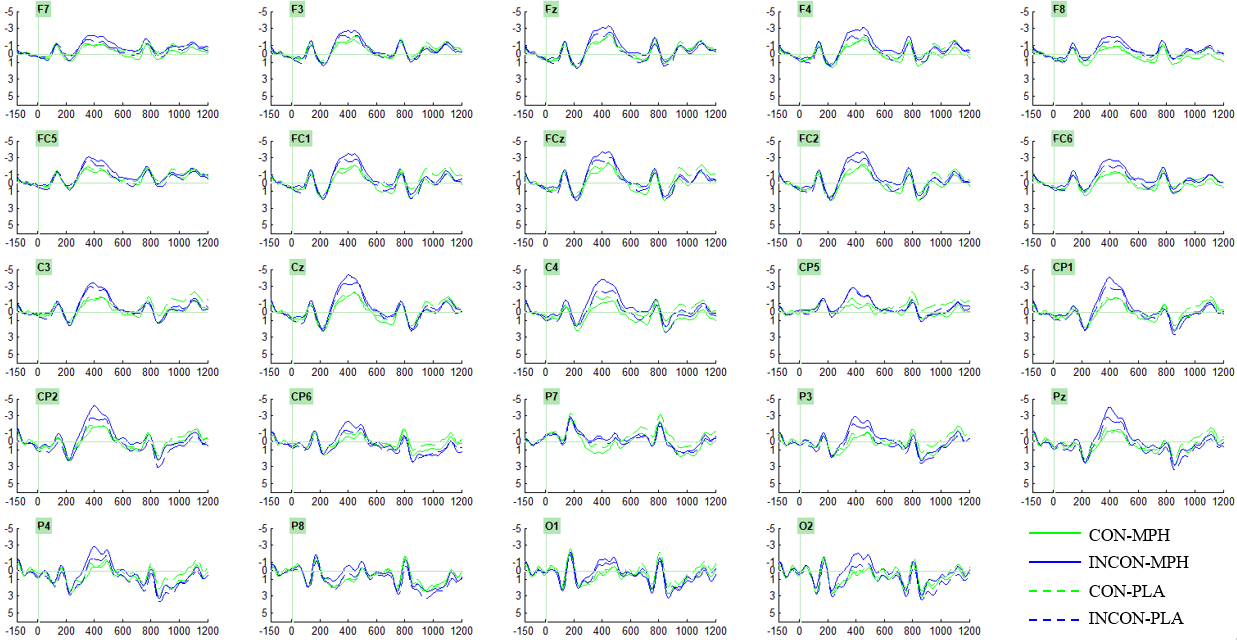


# Examine the effects of drug administration order in the ERPs

To examine the effect of drug administration order, we conducted a traditional 2 (MPH) × 2 (Congruence) × 2 (Anteriority) × 2 (Hemisphere) × 2 (Order) mixed factorial repeated ANOVAs on the EEG data. Overall, drug administration order did not interact with participants' EEG response in any pre-defined time window. The discrepancy between behavioral and EEG results was not surprising given that EEG response did not always couple with behavioral measurements (Brown & Hagoort, 1993). EEG recording provides a more sensitive measurement for online sentence processing to reveal the cognition change that were not evidenced in overt offline behavioral measures (Holcomb, Grainger, & O'rourke, 2002; Küper & Heil, 2009; Kutas & Federmeier, 2011). More importantly, it should be noted that in the behavioral analyses, we analyzed participants' offline response to perceptual violation (i.e., font size change detection) in the *Font-Size* task, while the EEG data always reflected participants' online response to the semantic manipulation. Therefore, the behavioral and EEG data were not expected to be fully consistent in the first place.

# Correlations between the MPH-induced changes in the behavioral data and the EEG data.

| Task | | Semantic Task | | | | | Font-size Task | | |
| --- | --- | --- | --- | --- | --- | --- | --- | --- | --- |
|  |  | d' | RT | Pre-N400 | N400 | LPC | d' | RT | N400 |
| Semantic | d’ |  | 0.036 | -0.180 | 0.019 | -0.047 | 0.191 | -0.231 | 0.274 |
|  | RT |  |  | -0.041 | -0.176 | -0.129 | 0.149 | -0.088 | -0.222 |
| Font-size | d’ |  |  | -0.019 | 0.227 | 0.100 |  | -0.142 | -0.239 |
|  | RT |  |  | -0.047 | -0.001 | 0.112 |  |  | 0.020 |

* *p* <.05. ** *p* < .001

The MPH-induced effects in the RTs were calculated as the differences between the incongruence effect (incongruent - congruent) in the MPH and the placebo conditions in each task. The MPH-induced effects in the d’ were calculated as the differences between d’ in the MPH and the placebo conditions in each task. The MPH-induced effects in the EEG responses were calculated as the differences between incongruency effect in the mean amplitude (incongruent - congruent) in the MPH and the placebo conditions in each task. It should be noted that although the MPH effect on the behavioral data interacted with the Order of drug administration, we examined the MPH effect in the entire group as a whole to increase the power and control for the family-wise error rate. Although the MPH effect on the behavioral data did not reach significance in certain group, this should not affect conducting the correlational tests on these data.

# Exploratory analysis: 10 ms step-wise analyses in the 80 – 160 ms time window

For exploratory purpose, we conducted a step-wise analysis by 10 ms between 80 ms to 160 ms on the MPH × semantic interaction, with a FDR control for multiple comparisons to examine the time window of the early effect observed in the study. The results are presented in Table D.

###### Table D. Results of the MPH × semantic in the early time window in the *Semantic* task

| Rank | Time window | *p-*uncorrected | *p-FDR* corrected |
| --- | --- | --- | --- |
| 1 | 130-140 | 0.0060* | 0.0063 |
| 2 | 140-150 | 0.0100* | 0.0125 |
| 3 | 120-130 | 0.0340 | 0.0188 |
| 4 | 110-120 | 0.0559 | 0.0250 |
| 5 | 150-160 | 0.0779 | 0.0313 |
| 6 | 100-110 | 0.1079 | 0.0375 |
| 7 | 90-100 | 0.1420 | 0.0438 |
| 8 | 80-90 | 0.3120 | 0.0500 |

Note. *p*-FDR corrected values are calculated as (k/m) × alpha. k is rank of the *p*-value while the *p*-values were listed in ascending order. m is the total number hypotheses tested. The null hypothesis was rejected if the uncorrected *p*-value was smaller than the corresponding *p*-*FDR* corrected value.

# Exploratory analysis: post-stimuli baseline results

Since an early EEG effect induced by MPH was observed in the semantic task, although no effect was observed prior to the critical word (i.e., on and after the immediately preceding adjective), we used a post-stimuli baseline of [0 - 100 ms] to control for the early differences after critical word onset and examined whether the later effects persisted. The N400 and the LPC effect persisted in both the *Semantic* and the *Font-size* tasks, but neither the MPH-induced effects nor their relation with WM capacity remained significant. From these analyses, we could not fully rule out the possibility that the MPH-induced change on N400 and LPC was a carry-over from the early time window between 100 - 150 ms.

# ERP effects on the adjective phrase

As the MPH effect showed up in the early time window on the critical noun, to make sure that such effect was not a carry-over from the previous word, we conducted permutation tests on the immediate preceding adjective of the critical noun (e.g., " *slimme/*zoute* " from the above example) with the same procedure. Results from the permutation tests showed that there was neither semantic congruence effect nor MPH-related effects in any time window on the adjective (as shown in Figure F). Therefore, the early effect observed on the noun phrase in the semantic task could not be attributed to the processing differences on the previous word.


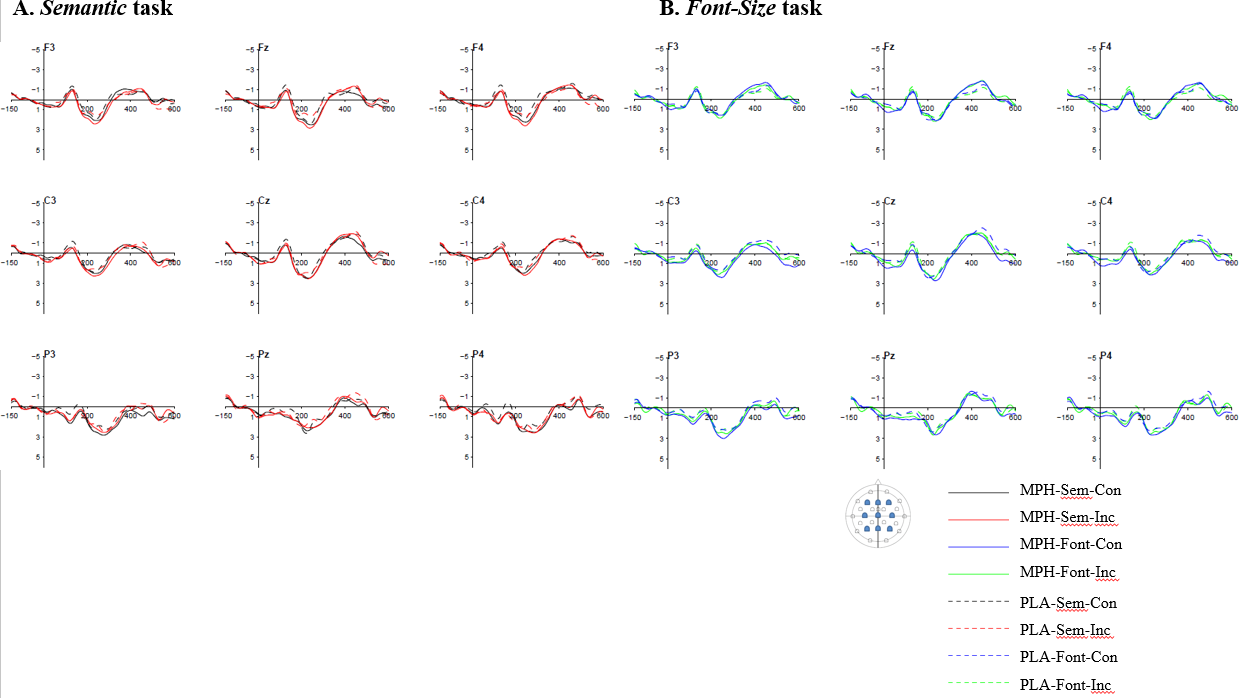


###### Figure F. Grand-averaged amplitudes of semantic congruent and incongruent sentences in the MPH and the placebo conditons, in the Semantic and the Font-Size tasks on the adjective (n-1 word to the critical noun). The negativity is plotted upward

# Relationship of MPH-induced changes on semantic processing to participants' baseline performance

As shown in Table G1, most participants' baseline performance of WM and language proficiency was within the normal range of the age-matched population (Neger et al., 2015; Tan, Martin, & Van Dyke, 2017). Participants’ spontaneous eye blink rate (SEBR) were also within the normal range in both placebo and MPH sessions (Doughty & Naase, 2006). Consistent with previous findings (Jongkees & Colzato, 2016), we found that participants with higher impulsivity as measured by the ADHD scale showed elevated SEBR, *r* = .40, *p* = .01. However, although the changes in participants' cardiovascular parameters indicated that our MPH manipulation is effective, we did not observe a significant systematic change in their SEBR after taking MPH, *t* (1. 41) = -.97, *p* = .34, while their SEBR on MPH and placebo conditions were highly correlated, *r* = .56, *p* < .001. The lack of MPH effect on SEBR was not surprising given that the results from animal models suggested that indirect DA agonists such as MPH did not affect SEBR (Kleven & Koek, 1996). It was still unclear whether MPH selectively affect striatal DA production.

###### Table G1. Measurement of the basic cognitive functions and spontaneous eye blink rate

| Capacity | Measurements | Mean | Range |
| --- | --- | --- | --- |
| WM | Reading span | 40.8/75 (16.0) | 7 - 72 |
|  | Operation span | 48.1/75 (18.1) | 6 - 75 |
| Processing speed | Box completion (s) | 61.8 (16.5) | 37.8 - 115.0 |
| Language proficiency | Language proficiency (Dutch version) | 55.8/80 (9.5) | 34 - 75 |
| ADHD | Inattention | 1.65 (.33) | 1.09 - 2.27 |
|  | Hyperactivity-impulsivity | 1.81 (.42) | 1.17 - 3.00 |
| SEBR (count/min) | (Placebo condition) | 11.7 (9.9) | 2.0 - 27.8 |
|  | (MPH condition) | 10.6 (6.9) | 2.8 - 37.2 |

Note.

1. The values in the parentheses represent standard deviations.
2. For the reading span and operation span tasks, the scores were calculated using traditional absolute scoring methods. Each participant’s score was the sum of all perfectly recalled sets and the set size varied between 3 - 7 items in the experiments. A latent variable of WM capacity was calculated from the two span tasks for further analysis. The latent variable provides a more reliable and robust measure than a single measure for a given construct (Nunnally, Bernstein, & Berge, 1967).
3. In the language proficiency task, participants had to fill in 40 blanks with content words in three independent paragraphs within 5 min to make each paragraph sensible. For each content word, two independent raters rated it on whether the word matched the grammatical (1 point) and semantic (1 point) structure of the sentence. Rating from the two raters were highly consistent, *r* = .95, *p* < .001. The average rating from the two raters was computed as the participants' score.
4. To access participants’ inattention and hyperactive-impulsivity characteristics, they were required to rate their own behavior on an ADHD rating scale with 23 items ("0" never/very rare to "3" very often). The average scores for inattention and hyperactive-impulsivity were computed separately. Participants' inattention and hyperactive-impulsivity scores were related (*r* = .42, *p* = .006). The average score of these two sub-scales was computed to index participant’s degree of inattention/hyperaction,

To examine whether any of the MPH-induced changes on semantic processing were modulated by individuals’ baseline performances, participants’ baseline measures were related to the MPH-induced changes on their ERP responses. The ERP differences were calculated by subtracting the semantic congruency effect (i.e., incongruent - congruent) in the placebo condition from that in the MPH condition, for the *Semantic* and the *Font-Size* judgment tasks separately. Then Pearson product-moment correlation was conducted between the ERP differences and individuals’ baseline performance (see Table G2 for the correlations among the baseline performance tests). For exploratory purpose, we did not correct for the multiple comparisons, but we have restrained our correlational analyses to the significant interactions in corresponding time windows. Correlations between the baseline measures and the N400/LPC effects were also reported because some previous studies suggested that the size of N400/LPC effects could be predicted by individuals’ WM capacity (Federmeier & Kutas, 2005; Federmeier, McLennan, Ochoa, & Kutas, 2002; Fiebach, Schlesewsky, & Friederici, 2001; Van Petten, Weckerly, McIsaac, & Kutas, 1997), but were of less interests.

###### Table G2. Correlations among the individual differences measures

|  | Language | Box | ADHD | Blink_PLA | Blink_MPH |
| --- | --- | --- | --- | --- | --- |
| WM | .343* | .143 | -.131 | -.088 | .089 |
| Language |  | .074 | -.024 | -.090 | -.066 |
| Box |  |  | .132 | -.102 | -.160 |
| ADHD |  |  |  | .320* | -.034 |
| Blink_PLA |  |  |  |  | .563** |

* *p* < .05; ** *p* < .01

As shown in Table G3 and Figure G, the results demonstrated that individuals with better WM capacity showed smaller change in the N400 effect when they were on MPH than placebo (*r* = -.421, *p* = .006). Further analyses showed that this was a result of a smaller increase of N400 amplitude in the congruent condition in the individuals with high WM capacity (*r* = .418, *p* = .006). These results suggest that MPH have a greater effect on intentional semantic processing in the individuals with low WM span. These were consistent with previous finding that the impact of MPH was modulated by the baseline characteristics of individual participants (Cools & D'Esposito, 2011b; Frank, Samanta, Moustafa, & Sherman, 2007; Mehta et al., 2000; van der Schaaf et al., 2013). However, the early effect between 100 - 150 ms was not related to any IDs measures. In addition, we replicated previous findings that individuals with high WM capacity tended to show a reduced N400 effect and increased LPC effect when they were instructed to do a purposeful semantic processing in the placebo conditions (Federmeier & Kutas, 2005; Federmeier et al., 2002; Fiebach et al., 2001; Vos, Gunter, Schriefers, & Friederici, 2001). Last, in the placebo condition, participants' SEBR was a good predictor for their N400 effect size in the semantic task, with higher SEBR related with greater N400 effect. This is in line with the clinical findings that schizophrenia patients, who had an elevated SEBR (Karson, 1983), tended to show greater N400 than healthy controls (Kiang, Kutas, Light, & Braff, 2008; Ryu et al., 2012), although there was few direct evidence about the relation of SEBR-N400 in the healthy population at this time.

###### Table G3. Correlations between the ERP effects and participants' baseline performance

|  | Semantic Task | | | | | | | Font size Task | | |
| --- | --- | --- | --- | --- | --- | --- | --- | --- | --- | --- |
| Time (ms) | 100 - 150 | 250-350 | 250-500 | | 550-900 | 550 - 1200 | | 250 - 500 | | |
|  | MPH × CON | MPH × CON | N400  (PLA) | N400  (MPH) | MPH × CON | LPC  (PLA) | LPC  (MPH) | MPH × CON | N400  (PLA) | N400  (MPH) |
| WM | -.293^a^ | **-.421**** | .467** | .006 | -.263 | .385* | .139 | .156 | -.045 | .182 |
| Language | -.287^b^ | -.272^c^ | .083 | -.174 | -.147 | .229 | .084 | -.032 | -.092 | -.063 |
| Box | -.129 | -.185 | .220 | .223 | -.126 | .141 | .190 | .161 | -.100 | .182 |
| ADHD | -.018 | .171 | -.053 | .040 | .179 | -.068 | .221 | -.039 | .107 | .071 |
| Blink_PLA | .074 | .089 | -.339* | -.171 | .238 | -.224 | .094 | .087 | .193 | .234 |
| Blink_MPH | -.044 | -.127 | -.086 | -.141 | -.079 | .107 | .024 | -.178 | .132 | -.129 |

* *p* < .05; ** *p* < .01

a. *p* = .060; b. *p* = .069; c. *p* = .085


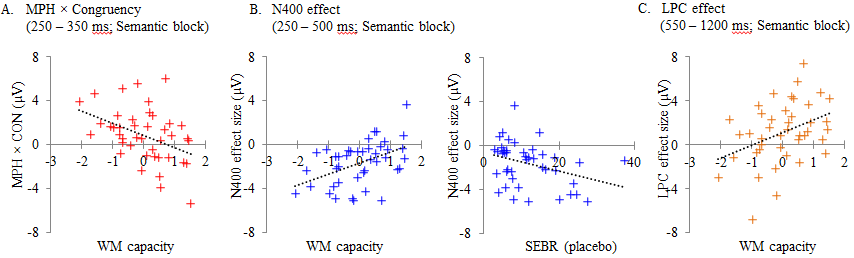


###### Figure G. Modulation effects of WM capacity and SEBR on MPH-induced change on semantic processing. In Fig. 5A, the MPH × Congruence interaction was calculated as the difference of N400 effect size between MPH and placebo conditions (i.e., N400_MPH_ – N400_placebo_). Fig. 5 B & C were the EEG response from the placebo conditions.

# Exploratory analysis: sentence with early vs. late position

To further investigate the underlying mechanism of the MPH-induced change on EEG response, we subdivided our sentence materials into two subsets based on the ordinal position of the critical word within each sentence. Previous findings have shown that as sentence unfolds over time, the magnitude of N400 response in the congruent sentences is systematically reduced with increasing word position because of increasingly semantic constraints, while the N400 response did not vary significantly in the incongruent sentences (Van Petten & Kutas, 1990, 1991). Therefore, taking the ordinal position of the critical word into consideration would potentially help us better understand the underlying mechanism of the MPH-induced change on the N400, and disentangle the MPH-induced change on N100-150 and N400 effects.

In the current experiment, in about 40% (143/360) of the sentences, semantic incongruence occurred in the beginning of the sentence and the critical noun was always the 3^rd^ word in the sentence pair (early-position condition), such as "*The blue/*sour train ...*", while in about 60% of the sentences, the critical noun occurred in a later position (late-position condition; mean = 8.6^th^ word, SD = 1.7), such as "*Mary travelled last weekend with the blue/*sour train ...*". A separate group of 37 participants who did not participant in the experiment were recruited to rate the congruence of the sentence right after reading the critical noun (1= not sensible at all; 7 = very sensible), before completed reading the entire sentence. Results showed that the later the critical noun’s position, the higher rating the congruence sentences received (*r* (36) = .13, *p* = .011), while there was no correlation between the critical noun’s position and the rating of incongruent sentences (*r* (36) = -.07, *p* = .19).

We hypothesized that the MPH-induced change on N100-150 effect in the semantic task might only manifest in the late-position condition, in which the semantic constraints were higher and thus lead to an earlier occurrence of the congruence effect on the N100-150. However, it should be noted that the limited and unbalanced number of trails between the early- and late-position conditions constrained the interpretability of the results. After subdividing the materials, there were on average 14.7 (SD = 0.7) sentences in each condition (MPH × Congruence × Task) in the early-position condition, and on average 23.6 (SD = 0.6) sentences in each condition in the late-position condition. Therefore, the results were discussed for exploratory purpose only.

As shown in Figure H, the pattern of the EEG results in both the early- and the late-position condition were generally consistent with the results based on all sentences. In the *Semantic* task, MPH tended to induce a reduced N100 and N400 effects and an increased LPC effect, while in the *Font-size* task, MPH tended to induce an increased N400 effect. In the *Semantic* task, results from the cluster-based permutation analysis confirmed our prediction that in the late-position condition only, there was an interaction of MPH × Congruence between 100 - 150 ms (*p* = .048). Planned comparison revealed that MPH induced a negative deflection in the semantic congruent condition than placebo (*p* = .01). However, there was no MPH-induced interaction in any other time window. In the *Font-size* task, there was no MPH-induced significant interaction in any time window (*p*s > .210)

On the other hand, in the early-position condition, there was no MPH-induced effect between 100 - 150 ms. Instead, there was an interaction of MPH × Congruence in both the *Semantic* and the *Font-size* tasks in the 250 - 500 ms and the 900 - 1200 time windows (*p*s < .05). In the *Semantic* task, the N400 effect only occurred in the placebo condition, while it disappeared in the MPH condition. Planned comparison between MPH and placebo conditions revealed that MPH induced a greater negative deflection between 250 –500 ms in the semantic congruent condition than the placebo condition (*p* = .004) ^[[1]](#footnote-1)^, while there was no difference in the semantic incongruent condition (*p* = 1.0). On the other hand, the LPC effect only occurred on MPH (*p* = .008) but not on the placebo condition (*p* = .180). No further comparisons reached significant in either congruent or incongruent conditions separately as in the previous analyses with all the items. In the *Font-size* task, between 250 - 1200 ms, there was a congruence effect while participants were on MPH as indexed by a sustained negativity (*p* = .042) with a central-parietal distribution, while there was no congruence effect on placebo (*p*s > .09)^[[2]](#footnote-2)^. The sustained negative effect might reflect a continuous semantic processing difficulty (Kos et al., 2012).

Taken together, our exploratory analysis revealed a somewhat different pattern of the MPH effect on semantic processing when the semantic incongruence occurred in the early- and the late-position conditions. When semantic constraint was relative high (i.e., late-position condition), MPH induced a smaller reduction of the N100-150 response on semantic congruent condition if semantic processing is task-relevant. When semantic constraint was relative low (i.e., early-position condition), MPH induced a greater N400 response on semantic congruent condition and a greater LPC effect than placebo when semantic processing was task-relevant. When semantic processing was task-irrelevant, taking MPH lead to a greater sustained negativity on semantic incongruent condition.


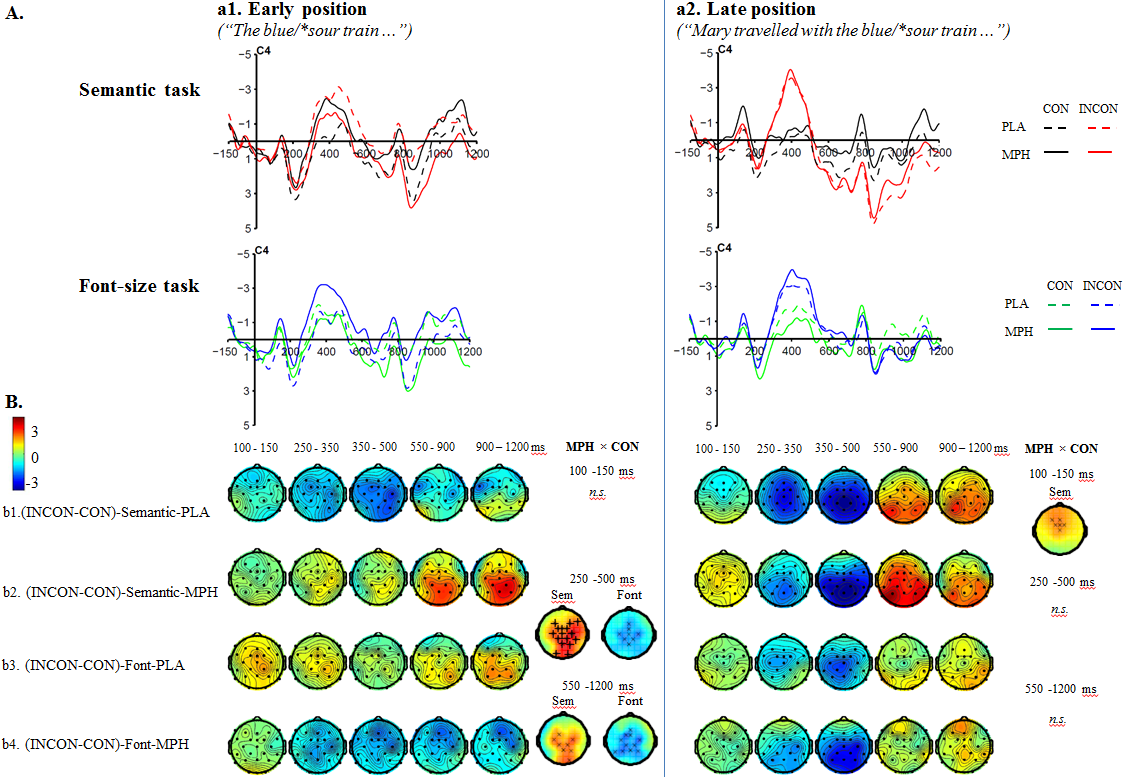


###### Figure H. Comparisons between the early- and late-position conditions. (A) Waveforms at a representative electrode (C4) in each condition in the *Semantic* and the *Font-size* tasks. The negativity is plotted upward. For illustrative purpose only, a 15 Hz low-pass filter has been applied on the waveforms. (B) Scalp distributions of the differences between semantically congruent and incongruent conditions on MPH and placebo in *Semantic* and the *Font-size* tasks. The electrodes that were included in the significant cluster of MPH × Congruence interaction were plotted as well. The positive interaction showed here suggested that the mean amplitude difference between congruent and incongruent (INC - CON) conditions was more positive (or less negative) in the MPH than the placebo condition, and the negative interaction showed the reversed pattern.

1. All the effects discussed in this paragraph were significant in both early and late time windows of the N400 and the LPC time windows. Therefore, we collapsed over the early and late time windows for N400 and LPC. [↑](#footnote-ref-1)
2. Or in another word, MPH induced a sustained negative deflection in the semantic incongruent condition than placebo (p = .044), while there was no reliable MPH effect in the semantic congruent condition (p = .336). [↑](#footnote-ref-2)
